# Explorative Imaging and Its Implementation at the FleX-ray Laboratory

**DOI:** 10.3390/jimaging6040018

**Published:** 2020-04-02

**Authors:** Sophia Bethany Coban, Felix Lucka, Willem Jan Palenstijn, Denis Van Loo, Kees Joost Batenburg

**Affiliations:** 1 Centrum Wiskunde & Informatica, Science Park 123, 1098 XG Amsterdam, The Netherlands; felix.lucka@cwi.nl (F.L.); willem.jan.palenstijn@cwi.nl (W.J.P.); joost.batenburg@cwi.nl (K.J.B.); 2 Centre for Medical Image Computing, University College London, London WC1E 6BT, UK; 3 TESCAN-XRE NV, Bollebergen 2B/bus 1, 9052 Ghent, Belgium; denis.vanloo@tescan.com; 4 Leiden Institute of Advanced Computer Science, Niels Bohrweg 1, 2333 CA Leiden, The Netherlands

**Keywords:** computed tomography, novel imaging systems, tomographic data acquisition, live image reconstruction

## Abstract

In tomographic imaging, the traditional process consists of an expert and an operator collecting data, the expert working on the reconstructed slices and drawing conclusions. The quality of reconstructions depends heavily on the quality of the collected data, except that, in the traditional process of imaging, the expert has very little influence over the acquisition parameters, experimental plan or the collected data. It is often the case that the expert has to draw limited conclusions from the reconstructions, or adapt a research question to data available. This method of imaging is static and sequential, and limits the potential of tomography as a research tool. In this paper, we propose a more dynamic process of imaging where experiments are tailored around a sample or the research question; intermediate reconstructions and analysis are available almost instantaneously, and expert has input at any stage of the process (including during acquisition) to improve acquisition or image reconstruction. Through various applications of 2D, 3D and dynamic 3D imaging at the FleX-ray Laboratory, we present the unexpected journey of exploration a research question undergoes, and the surprising benefits it yields.

## 1. Introduction

In many applications in research as well as in the industry, computed tomography (CT) is becoming the standard tool to carry out nondestructive inspection of the interiors or testing the properties of an object [1,2,3,4,5]. The common practice in tomographic data acquisition is that an expert with a research question acquires data with the help of an operator. It is the operator who decides on acquisition parameters and makes adjustments depending on the object or experimental limitations. Further, the experimental design is based on the operator’s previous experiences or is restricted to hardware limitations. The entire process is static and sequential in the sense that an experimental plan is drawn up, data are acquired and reconstructed, and results are concluded. Any changes to the experimental plan are time-consuming, as are any follow ups between the expert and the operator during acquisition to ensure data collected truly answers the research question. There is also no guarantee that the adjustments or fine-tuning made by the operator do not influence the final analysis and therefore the conclusion of the research.

A clear advantage we miss out on in the traditional process of acquiring data is the expert’s unique knowledge in their research topic. When the expert and the operator decide on an experimental plan, it is often the case that the research question is not well explored, and during the acquisition the expert is severely limited by time and therefore has few opportunities to investigate a problem further. This is especially a problem at synchrotrons, where the experts have to stick to a tight schedule. This is currently being improved through efficient data post-processing and reconstruction implementations at various synchrotrons in order to deliver results within minutes in-between scans, e.g., SAVU at Diamond Light Source [6]. In a laboratory setting, the issue becomes that the assigned operator often collects data in a routine manner with no exposure to the expert’s field or understanding of the research question. In the end, the expert becomes restricted to the collected data, and tries to conclude any novel understanding from it until such a time they can perform experiments again (if at all).

**Figure 1:**
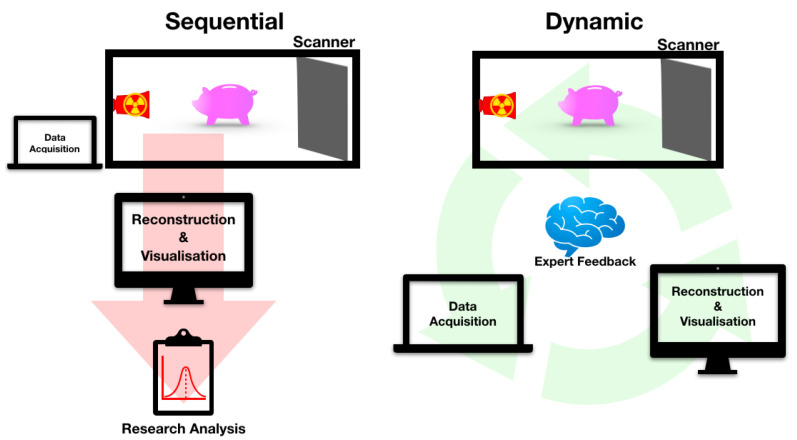
An illustration of the basics of explorative imaging (dynamic) in a tomography setting, with the traditional process of imaging (sequential) included for comparison.

In the last decade, we witnessed rapid advancements that had direct impact in the field of imaging: We now have access to higher computational power; hardware components of a CT scanner have greatly improved, especially with more reliable X-ray sources and cameras with higher shutter speed; data transfer is now faster and more stable; and we have developed more sophisticated image reconstruction algorithms. In addition, we as a community can benefit more by revising this methodology of acquiring and working with tomographic data. In this paper, we propose a more dynamic process of acquiring and processing data, where experiments are tailored around the sample or the research question; intermediate reconstructions and analysis are available almost instantaneously, and expert has input at any stage of the process to improve acquisition or image reconstruction. This process works in a loop, in an iterative manner and is illustrated in Figure 1. Due to the nature of exploring the sample (or the research question) within a loop alongside an expert, we refer to this as explorative imaging. We note that, while some operators do follow the idea of explorative imaging naturally, typically it is either followed partly, limited by software-hardware integration, or they simply do not conceptualise exploration in the process of imaging. In this paper, we aim to clearly define what explorative imaging entails, and highlight through case studies any benefits it brings over the sequential imaging.

Creating this interaction between the operator and expert requires detailed and timely 3D insight into the object while carrying out the scans. For the dynamic process to function as envisioned, we need a highly flexible hardware setup that can be easily customised; a fast and reliable data streaming service; and a reconstruction platform that is able to output volumetric information rapidly as the data stream starts (i.e., almost-real time). In addition, a complete framework for explorative imaging requires a reliable software and hardware integration, and a visualisation tool connected to the reconstruction platform. Such framework is brought together in the FleX-ray Laboratory, which we introduce in the next section. In Section 3, we exercise three case studies to demonstrate the practicality of explorative imaging where the unexpected information we discover led to additional insights. We finalise the paper with discussion and our vision for the future of explorative imaging.

## 2. FleX-ray Laboratory

**Figure 2:**
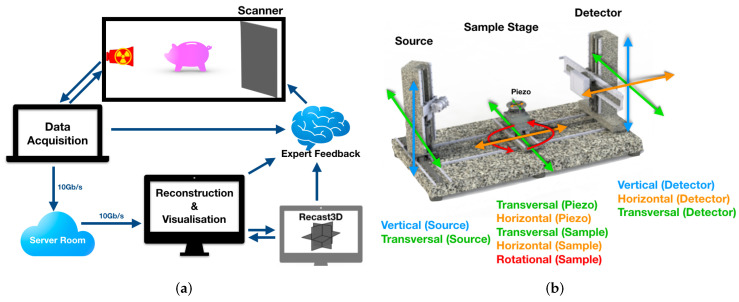
The FleX-ray Laboratory setup sketched in detail in (**a**), and the scanner motors within the FleX-ray scanner shown in (**b**).

We established the FleX-ray Laboratory through the collaboration of CWI, TESCAN-XRE NV, Nikhef and Amsterdam Scientific Instruments (ASI), and, with the combined work and expertise of the authors, developed the framework for explorative imaging (illustrated in Figure 2a). The FleX-ray scanner is a custom built, highly flexible lab μCT system with 10 motors that are individually programmable. A diagram of the FleX-ray scanner with its motors is in Figure 2b.

### 2.1. A Custom Built Scanner

The apparatus consists of a cone-beam microfocus X-ray point source with energy range of 20–90 kV, and offers a focal spot size of 17 μm. The source projects polychromatic X-ray beams onto a CMOS (complementary metal-oxide semiconductor) detector with CsI(Tl) scintillator (Dexela1512NDT, see [7]), which is a 1944×1536 pixels, 14-bit, flat detector panel. The physical size of the detector is 14.59 cm × 11.49 cm, and the pixel size at the detector is 74.8 μm.

**Figure 3:**
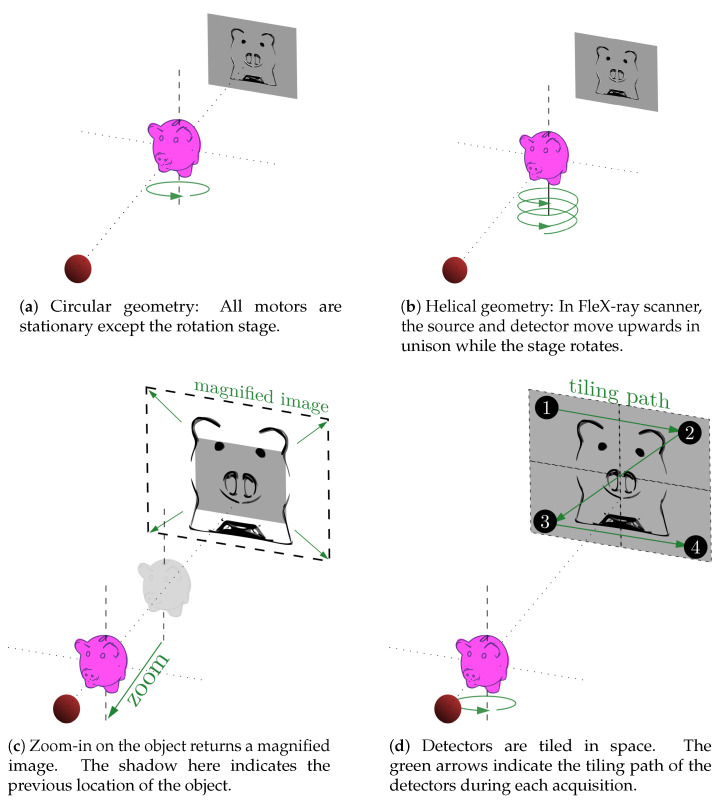
The top row illustrates typical scan geometries and the bottom row the process of spatial tiling of the detector in FleX-ray scanner.

Due to the flexibility of the apparatus, it is possible to emulate many other scanning geometries (e.g., helical or laminography), or take larger projections (i.e., higher resolution images) via spatial tiling of the detector. Example illustrations are given in Figure 3. For example, while the default geometry for the scanner is the circular geometry (i.e., all motors are stationary except the rotational stage, which can only rotate on a horizontal plane), we can still perform a helical geometry scan. This is done by moving the source and the detector upwards at a given speed simultaneously, while the stage rotates as in the circular geometry (see Figure 3b). Similarly, if we want to perform a higher resolution scan (i.e., move the object closer to the source as in Figure 3c) and capture the full-size data (now magnified), we can program the motors so that the detector follows a tiling grid, where at each point circular tomographic data are collected. The green arrows on the detector plane in Figure 3d indicate the tiling path, with the numbers as the grid points for each tile. The tile radiographs taken at each point can be later stitched to create a complete projection larger than the detector itself. The stitched data is then used to achieve a full reconstruction of the sample at a higher spatial resolution. The motors can be programmed for the full duration of a scan, or can be interrupted to take on new commands from the operator during data acquisition. In addition to programmable motors, we can also change tube or camera settings during an experiment for better suited data acquisition around an object or to follow an unusual experimental plan. The sample stage offers a continuous rotation, and the data are discretised by the exposure rate of the camera at that time, and are then stored as 16-bit tiff files in the given directory.

The scanner comes with a Windows executable named Acquila (TESCAN-XRE), which is a user-friendly program to command the machine to perform template scans (such as circular cone-beam, tiled, stacked, etc.). In our setup, this program functions as a server in which we communicate with individual motors, and feed specific commands to perform tailored experiments. Along with its flexibility, what makes the FleX-ray Laboratory a suitable apparatus for explorative imaging is its integration of hardware with the internal data streaming service (with the capacity of 10 Gb/s) and the software developed in-house.

### 2.2. Software Details

Another crucial step in the explorative imaging framework is the almost-real time tomography, for which we utilise our open-source software, Recast3D. This software provides reconstruction of arbitrary slices in each axis in 3D at any requested time. These slices can be tilted and moved to produce almost instant slice reconstructions as soon as data arrive via the stream. This is referred to as quasi-3D reconstruction, and is made possible via exploiting the mathematical properties of filtered backprojection-type methods, explained in more detail in [8]. By reconstructing and transferring only the visualised 2D slices, we avoid the heavy computational and network requirements of full 3D reconstructions. In its core, Recast3D uses the ASTRA toolbox, a software toolbox for the development of advanced reconstruction algorithms with the use of parallel forward and backprojection implementations for GPUs [9]. The acquisition parameters are passed to Recast3D via a wrapper, now as part of the FleXbox toolbox [10]. This toolbox also provides a selection of advanced iterative reconstruction methods to perform high-quality reconstructions after a scan has been completed, if the real-time FDK reconstructions provided by Recast3D are not sufficient. The authors also produced a simple scripting program, where an expert can create custom scripts for FleX-ray scanner to make full use of the flexibility of the system and communicate with individual motors via simple commands prior to or during acquisition.

## 3. Explorative Imaging in Action

### 3.1. Case Study I: Cultural Heritage

Restoration, conservation and provenance of cultural heritage objects is of great interest in the fields of art, history and social sciences. Studying cultural heritage objects helps us understand the methods, tools and materials available to a particular society at that point in time. It also reveals information such as how societies functioned, how social classes were defined, economic exchange and trading between regions across the globe, and daily lives, language and diets of ancient cultures [11,12,13].

Working with objects of this nature can be challenging as the samples require a flexible setting and tailored experimental plans to accommodate unusual research questions. Experts often have to adhere to limitations in a laboratory setting and the experiments become too expensive to conduct elaborately at synchrotrons. Further, particularly in a laboratory CT environment, there is no opportunity for the expert to directly use their knowledge to influence experiments based on any immediate results or status of data acquisition. Here, we present a case where we continuously improve the quality of acquired data (and thus the research question) by offering intermediate solutions to the expert and incorporating the feedback immediately back into the loop. To capture the conversation between the operator and the expert, this study is presented in a dialogue-like format, where the italic subheadings are questions asked directly by the expert, and the following text is a documentation of the joint effort to find an answer. It is worth nothing that the experiments, reconstructions and discussions for the next iteration for this sample took place in a single afternoon.

In this particular example, we study a terracotta sample, “the Torso” [14], from a series of mini sculptures from the Rijksmuseum collection [15] (pp. 294–297). These were originally believed to be practice models by Michelangelo for his bigger projects [16,17]. However, further study into the authenticity concluded that this claim was false, and instead attributed the items to a lesser known Dutch sculptor Johan Gregor van der Schardt.

**Figure 4:**
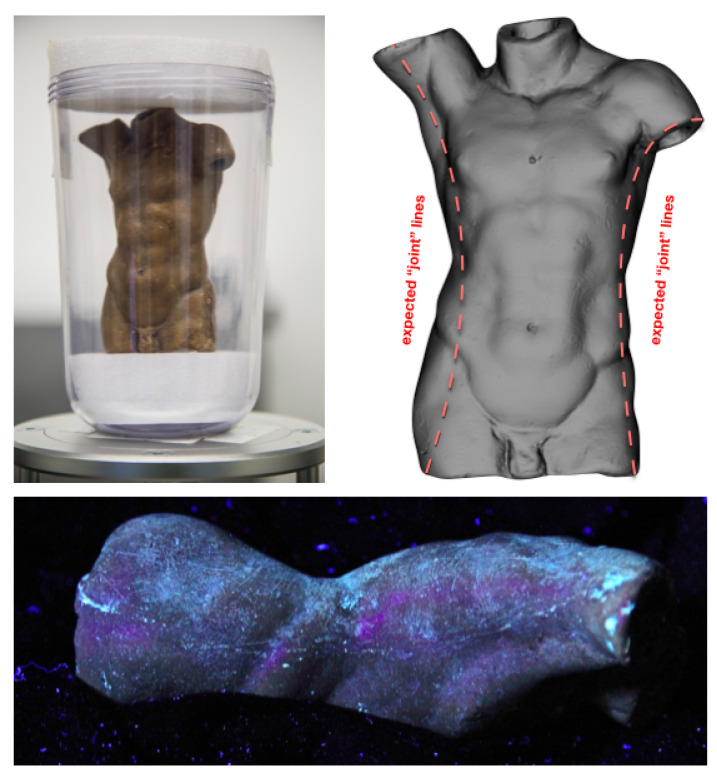
Sample mounted in perspex glass container on left; and a low-resolution rendering of the volume annotated with dashed lines along its side to show the expected “joint lines” on right. Bottom is one of the ultraviolet images taken of the sculpture, showing the joint line on one side; image was provided by the expert.

A reliable way of verifying the authenticity of these sculptures is to study the methods used to create them. The experts believed that this piece consisted of two moulds (front and back of the Torso) joined together. This is based on ultraviolet images of the sculpture, which showed thin, straight lines along each side of the object (see Figure 4, bottom). Thus, our initial task was to verify this claim.

*Can We Determine Any “Joint Lines” along the Sides of the Sculpture?*

We performed a fast, low resolution scan with the entire object in full view of the detector. The resolution was 136 μm${}^{2}$, and the total scan time was 15 min, with 1200 projections collected. The acquired data were immediately reconstructed and rendered for the expert to study. This scan was done to get a clear impression of the object overall, as well as answering the initial research question. Figure 4 (top left) shows the object mounted using a perspex glass container, situated in a bed of foam to dampen any vibrations that may arise from rotating the sample. Figure 4 (top right) also shows a rendering of the object after the initial scan, where the expected “joint lines” in the object are marked by dashed lines.

**Figure 5:**
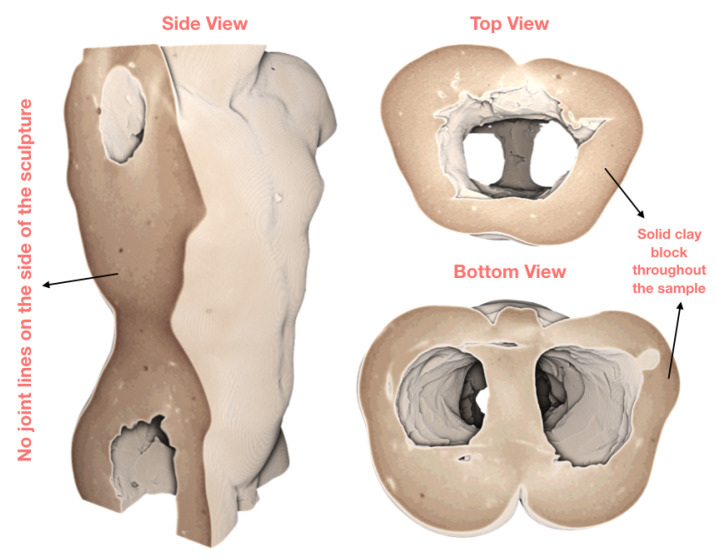
Despite the initial expectations, the volume renderings reveal no “joint lines” along the sides of the sample.

Figure 5 shows three views—right side, top and bottom of the volume, sliced inwards to reveal no joint lines within the walls of the statue. This suggests that the sample was fashioned from a solid block of clay. These renderings also showed rich texture on the inside of the sample, which took us to the next iteration in the loop.

*Can We Find Any Toolmarkings On The Inside?*

**Figure 6:**
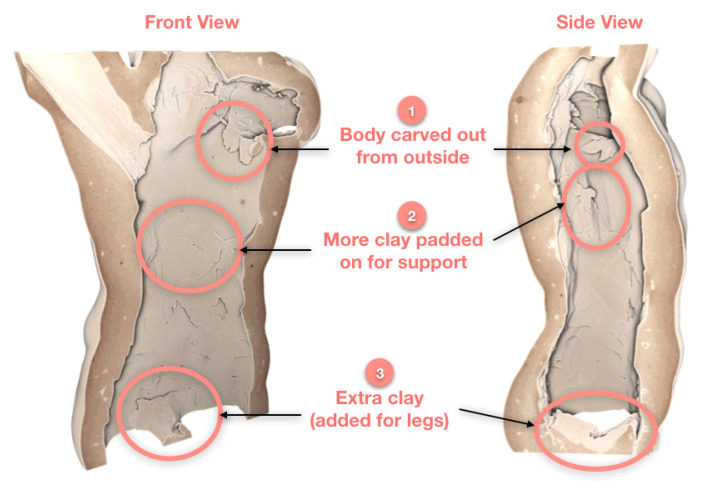
Marked locations highlight the techniques used to shape the hollow terracotta sample.

We studied the reconstruction in more detail, sectioning in from the front and further from the side of the volume. The renderings in Figure 6 are annotated with markers for various toolmarks or techniques observed.

Lack of joint lines means that the sample is made from a single block of clay, first moulded into its current shape. Markers 1 and 3 confirm that the sculpture was indeed carved out from the bottom and the top (through the arms and neck). Marker 2 shows that the sample was carved from the bottom and later reinforced with more clay to add support to the walls of the hollow statue. In addition, Marker 3 shows extra clay was added to join the bottom of the sample together to form the legs.

**Figure 7:**
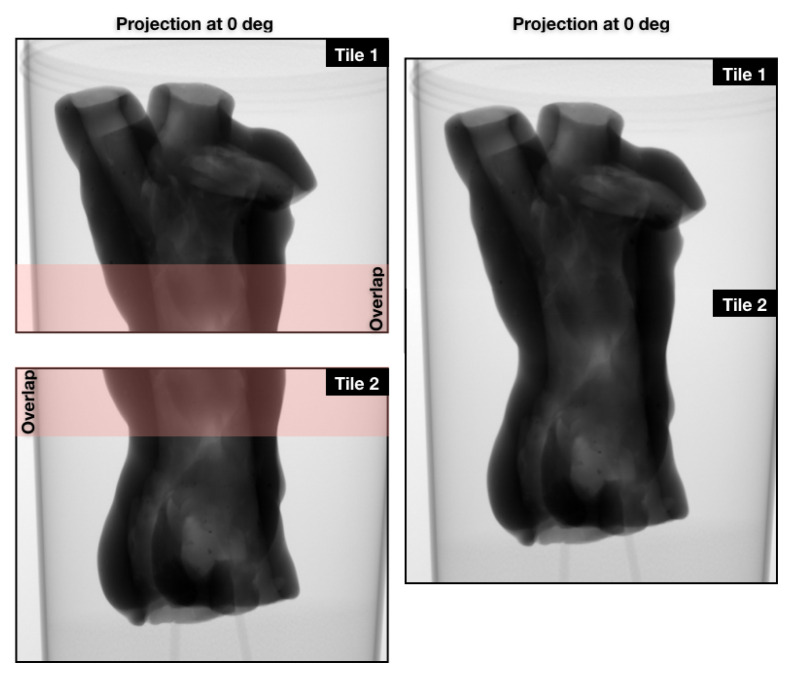
The tile radiographs on the left with the overlapping zone highlighted, and the stitched radiograph on the right.

**Figure 8:**
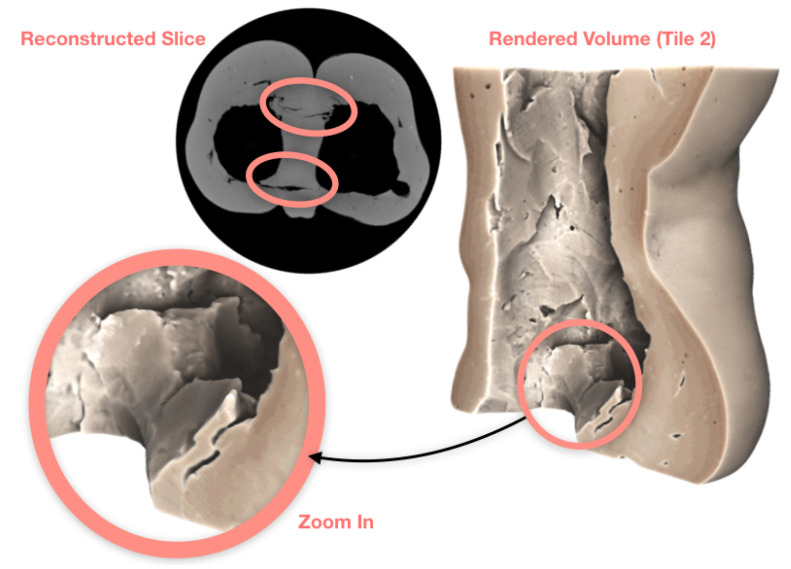
The joint lines with gaps at their largest. Gaps are circled both on the reconstructed cross-sectional slice and the volume rendering, including a zoom-in on the top of additional clay.

To study the joint lines around the additional clay (legs), we needed a higher resolution reconstruction. The objective was to extract more accurate information, such as the size of the gaps, how deep they are and how far along they run. We also needed more information from the middle section of the statue, where more clay was visibly padded on. We performed a scan with the object zoomed in by over a factor of two (i.e., using two vertical tiles of the detector). In tiled scans, we often have a buffering zone where two adjacent tiles overlap by a small margin. This ensures the registration of tiles and accurate stitching of the data. Figure 7 consists of two radiographs with the overlapping zone highlighted on the left, and the stitched version on the right. The tiled scan offers 50 μm${}^{2}$ pixel resolution, with 2000 projections and five times longer averaging for each projection. This increased the scan time to 55 min per tile. These two-tile data, and the previous single-tile data can be found in [18]. Additional photos of the object can be found in the Supplementary Materials. We include a reconstruction of a cross-sectional slice of the bottom tile in Figure 8, along with higher resolution rendering that show clearer texture and gaps along the joint lines. The volume rendering is sliced from the side to show the gaps at their largest in the joint lines, which decrease in size as we reach the physical bottom of the sample (the very bottom of the legs).

The motivation for inspecting the top tile was to identify the techniques the artist used to create the object. However, we observed an interesting feature that took us to the next iteration.

*Are Those Fingerprints?*

**Figure 9:**
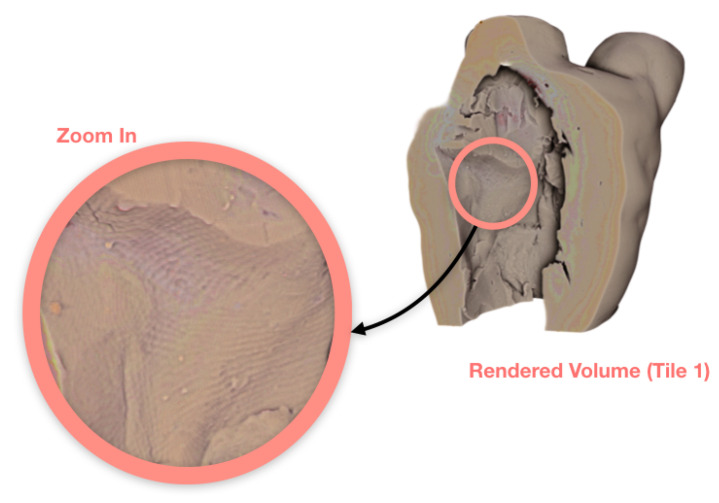
Distorted (suspected) fingerprints on the interior of the sample.

While inspecting the top tile, we noticed shapes that resembled fingerprints on the interior of the clay walls, an example is given in Figure 9. Expert suggested this could be a result of tapping, padding or dragging excess clay around with fingertips. This could also be an “aliasing” (or a “staircase”) effect due to the rendering, which in computer graphics is a distortion of curved lines that appear in a jagged or stair-step fashion in rendered volumes.

Although the lines do appear to mimic the curvature of a typical fingerprint as opposed to neat, pixelated staircases, the possibility of these being rendering effect cannot be ignored. This motivated a quick scan with similar acquisition settings (same spatial resolution), where we squeezed a lump of play-dough from the sides using the thumb, ring and index fingers.

*Can We Resolve Fingerprints in a Similar Experimental Setting?*

**Figure 10:**
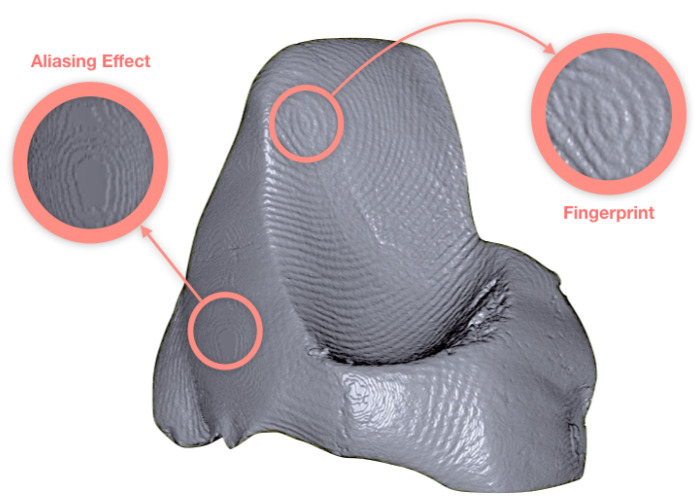
Fingerprints and the aliasing effects highlighted in the play-dough sample, in a scan comparable the terracotta samples.

We are indeed able to distinguish the fingerprints from aliasing effect. The squeezed facades clearly indicate jagged lines that move in an almost-spiralling manner as a fingerprint, whereas the untouched sides show the staircases descend towards the edges in a circular manner (see Figure 10).

This is an illustrative example to test whether we can distinguish a fingerprint from the aliasing of the 3D volume. The prints found inside the terracotta sample were more distorted than a simple aliasing effect, as we suspected the artist may have tapped on more clay or dragged fingers to remove excess material. Despite this, the expert believes a clear fingerprint can be extract and compared to those (if any) from other samples to accurately authenticate and establish the terracotta series.

This case is a great example of what impact an expert feedback can have on the acquisition and analysis of the collected data, and the path of exploration we take in each loop. What started as a simple question of “looking for joint lines” evolved further into better defined questions, and therefore a more concrete authentication technique of matching fingerprints. This would not have been possible in the sequential method of imaging where the expert would likely be absent from the acquisition or not been presented with intermediate reconstructions.

### 3.2. Case Study II: Ink Layers

We are presented with a manuscript documenting mathematical solutions to the famous textbook “De Cijfferinghe” (i.e., The Calculation) by Willem Bartjens. The dating in some chapters and consistent handwriting throughout suggest that the manuscript was produced between 1661 and 1664 by a single author. There are 18 signatures within the manuscript, all of which are crossed out. Thus, the identity of the author or the origin of the manuscript are unknown.

The ink used to cross out the name (or signature) of the author appear to be thicker and darker. Previous research by the owner (henceforth, the expert) concludes that both inks are “iron gall”, the standard ink formulation commonly used in Europe from the 5th until the early 20th century. Research also suggests that the two inks differ in their chemical compositions, with the original ink being higher in metal (due to higher concentration of iron). This information can be used to date inks [19,20,21]. In this case, the ink used to cross out author’s names was produced in 18th, 19th or possibly early 20th century. While this rules out the techniques of correlation and dating to familial connections, the use of X-rays to discover the name of the author is still plausible since the iron gall on paper has high contrast. Previous work in the literature for uncovering writings in old manuscripts exist (using X-ray fluorescence or phase contrast imaging in synchrotrons) [22,23,24]. In contrast to these studies, we have two levels of ink on top of paper, similar to the first two cases mentioned in [25] but a laboratory X-ray absorption imaging instead of a synchrotron X-ray fluorescence imaging. This led us to the question: Through a combination of filters applied to the X-ray spectrum and careful experimental planning, can we recover the name of the author? There is a direct correlation between the attenuation contrast and the chemical compositions of the inks (i.e., the differing iron content) [26]. In addition, iron gall is corrosive, meaning the original ink may have left a lasting impression on the paper despite the newer ink on top of it.

Prior to data acquisition, we considered the harmful effects of X-ray illumination on old scripts and inks. The literature suggests that radiating samples of this nature is safe up to 15 kGy [27,28]. In our laboratory, with typical settings, this amounts to approximately 300 h of scanning. As per our experimental plan, we concluded that the sample would be exposed to radiation no more than 20 h (the total exposure time was less than 6 h).

**Figure 11:**
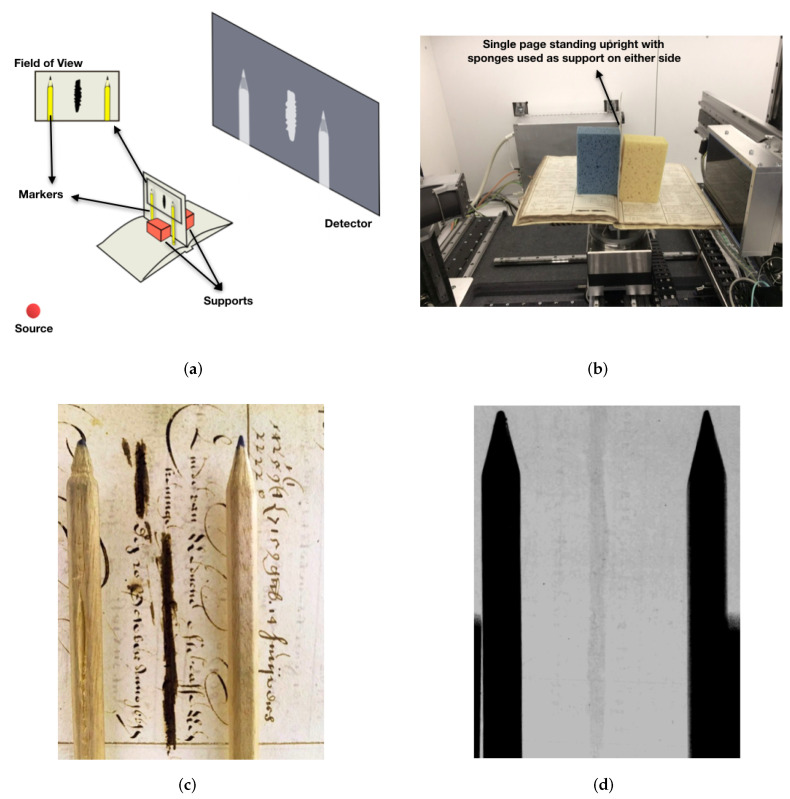
An illustration of the experimental setup is given in (**a**), with a photograph of the realisation inside the FleX-ray scanner in (**b**). An example field of view with a given magnification is shown in (**c**) with pencils used as markers, and the unprocessed radiograph of the same field of view in (**d**) with the sponges behind the scanned page acting as supports.

The mounting consisted of a solid base for the book to rest on, with a single page standing upright using soft supports (such as sponges). We also used pencils to act as markers for the orientation of the scribblings, since a sheet in the book could contain more than one signature, on either side. The following figure includes a diagram of the experimental setup and a photograph of its realisation (Figure 11a,b, respectively), as well as an instance of the scribbling while mounted (Figure 11c), and its unprocessed radiograph (Figure 11d). It is important to note that, in this experiment, only the tube and the detector moved up, down or sideways, and the sample was stationary at all times. The (unprocessed) radiographs shown in this study, as well as additional photographs of the manuscript, are included in the Supplementary Materials.

**Figure 12:**
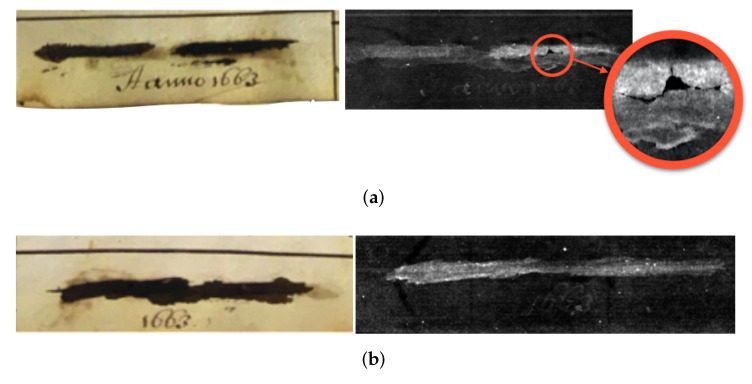
Two examples of the false positives with photographs of the scribblings on the left, and processed radiographs on the right. The top set in (**a**) indicate a partially torn page, and the bottom set in (**b**) show no detectable calligraphy in the post-processed image.

Due to the fragility of the object and the scribblings lying on a flat plane, we decided to take 2D radiographs of the 18 locations in the book. Our original plan of action was to take high quality radiographs (long shutter time, averaging and optimal exposure time) using various energy levels (10, 15 and 20 kV) with two different filters (aluminium and copper) as well as no filter. After image correction and basic processing techniques (erode, dilute, despeckle and denoise), we planned to extract and separate individual letters to correlate all instances of the scribblings. This method required automation, and eliminated the need of human interference or feedback. A limitation is that we relied heavily on the input data being precise enough, assuming all letters are extracted from all signatures. This proved to be a challenge once we discovered that some of these 18 instances were false positives, i.e., scribblings with no actual input or clear signature, or those that are scribbled so harshly that the paper itself is torn (undetectable to the naked eye). The expert feedback here was particularly useful in identifying the false positives, and saved scanning time in the long term. Some false positives were more obvious than others. The following figure shows two examples of this: Figure 12a is the page completely torn in the second section of the scribbling and Figure 12b is one that is showing no calligraphy following the same imaging process as the rest. The latter can be a result of the original signature being written too lightly that it made no imprint on the page itself, and as a result the scribbling that followed after completely wiped out the author’s name. This, as we later discovered, was common among the 18 locations.

**Figure 13:**
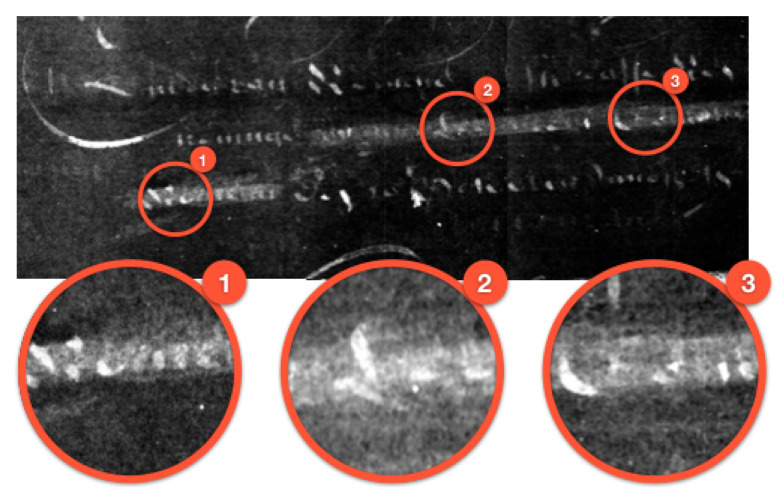
Zoom-in on the three initials of the signature.

This led us to revise our plan of correlating and averaging all signatures, and we instead focused on individual instances that we felt produced reliable data, one of which we include in Figure 13. For this particular case, and due to the size, we took two radiographs with a large overlap and stitched in post-process. Figure shows the final image along with zoomed-in windows on the initials of the author’s names. These windows are marked 2 and 3 for the initials of first and middle names, and the first is of the family name. The lettering at this point is somewhat clear but this still proved to be a guessing game to the untrained eye. During image processing, we were able to make suggestions such as *Lea* or *Lee* for the first name; *Cor* or *Coe* for the middle name; and finally *Mon* or *Rom* for the family name of the author.

Based on the findings in our experiments, along with previous research carried out by the expert, the author name was revealed to be Leendert Cornelisse Romeijn. Leendert is the first name, a seemingly common Christian name at the time. Cornellise is the middle name, a patronym used as a reference to author’s father, Cornelis (Cornelisse means Cornelis’ son). Finally, Romeijn is the family name, possibly a version of an original Christian name “Romein”, or one to mean “coming from Rome” or of the big gypsy family called “Roma”. The national archives contain a certain individual with the name Leendert Romeijn around the time the manuscript was written, who lived in Rotterdam and had six children. One of these offsprings was named Cornelis Romeijn, which aligns with the common tradition of naming the first son after the father-of-father (i.e., Leendert’s father, whose name we know from the book is Cornelis). Furthermore, Leendert lived in Rotterdam at a time when the city boomed with commerce and international shipping, another reason for a trader such as Leendert to study the Bartjens’ textbook and collect his solutions in a personal manuscript.

In this case study, we made great use of the flexibility of the FleX-ray scanner by constantly repositioning motors to better fit around a difficult and fragile object. Having the expert providing feedback at each stage of the process allowed a more refined method to be employed for the next stages. The feedback also saved time by determining false positives early in the experiment.

In addition, combination of various energy levels and flux for X-rays greatly improved the image processing and the final conclusion: we found especially in detecting false positives that the radiographs with varying energy levels confirmed the absence of the names. Thus, we believe these experiments can be repeated with a spectral detector, which would add another dimension of information and clarity.

### 3.3. Case Study III: Gas Bubble Travel Through Liquid

The FleX-ray Laboratory framework offers the capability of almost instant quasi-3D volume reconstructions (in the form of three cross-sectional slices), allowing the operator and the expert to track any structural changes in the sample. This creates an ideal environment for any in-situ or dynamic experiments as the slice reconstruction in three axes is available to the expert instantly, and any necessary changes can be made or intermediate results can be obtained while the data are acquired.

To achieve the speed required to capture the dynamics, the consensus is to acquire data at a synchrotron light source facility. This is due to the high speed for production of monochromatic X-rays as well as the rotational and frame capture speed made possible by the equipment at high resolution [29,30]. Selected works show the necessity of synchrotrons for time-resolved experiments, to capture, e.g., the interactions of a free-falling drop of liquid within a porous media to study fluid transfer in inter- and intra-grain domains [31]; the fracture process in a weak aluminium composite via in-situ tensile stress continuously applied [32]; and crystal nucleation and growth kinetics in natural lava via in-situ high temperature environment to study volcanic eruptions and lava viscosity [33]. The examples given here exhibit fast-dynamics changes in sample where changes happen in subseconds.

Conducting experiments at a synchrotron light source is costly and restricted to an allocated time period. This limits the amount of time an expert can spend on studying the acquired data before refining the next set of experiments. This motivated a shift in focus towards the laboratory-based X-ray CT systems (henceforth lab-CT), reducing (but of course not eliminating) these concerns. Here, the acquisition speed is limited by detectors with thick scintillators; the spatial resolution restricted, and the Poisson noise increased and the SNR decreased due to the lower flux of the X-ray tubes at high frame-rates. Despite these drawbacks leading to reduced temporal and spatial resolution compared to synchrotrons, fast dynamic 3D scans are made viable in a lab-CT environment via the use of advanced reconstruction algorithms such as iterative methods with noise-reducing [34,35,36,37] and structural model-based priors [38]; discretisation in image or gradient domains [39]; compressive sensing of limited data [40,41,42]; or direct methods with learned filters [43]. A comprehensive review and a timeline of trends in reconstruction methods for all purpose can be found in [44].

In all sources above, the reconstructions are done post-acquisition, and the experiments are adjusted depending on the data already acquired. In this section, we demonstrate our final component of the pipeline at the FleX-ray Laboratory by providing almost-instant reconstructions in time-resolved experiments performed on site. The lab-CT limitations mentioned above are still valid for our system. However, the collected data are instantly available as a reconstruction in arbitrarily selected slices in three axes, thus allowing the expert to incorporate immediate feedback into the experiment to adjust and refine the acquisition.

For this case study, we examine the behaviour of bubbles in a constrained space as bubble physics are important in many fields. For example in modelling magma flow, dynamics of bubble growth dictate changes in chemical and physical properties of magma, leading to clearer volcanic activity observations and more accurate models for eruption forecast [45,46,47]. Bubble size and distribution in liquid is studied to assess the quality of champagne or sparkling wine [48,49]. Similarly, bubble growth and coalescence in the fermentation process of breadmaking is studied to understand bread texture [50,51]. In material science, bubble growth in the curing of polymers induced by moisture is quantified for quality control [52]. Understanding and controlling bubble cavitation helps avoid pressure-driven shockwaves in hydraulic pumps or ship propellers, ensuring lower running and maintenance costs as well as noise reduction underwater. This is a vital aspect to aerospace and maritime engineering [53,54,55,56].

As an illustrative case for a dynamic experiment, we observe effervescent bubbles produced via the interaction of water-based gel with an aqueous medium. The setup consists of a store-bought denture cleaning tablet, placed at the bottom of a clear cylindrical plastic container. These tablets are typically designed to be fast-dissolving, and produce small and compact channels of bubbles. We use a denture cleaning tablet in particular as the dissolution time in water varies from 3 to 5 min, meaning the bubbles are produced at a slower rate. In addition, we use a store-bought water-based gel instead of water to slow down the bubble displacement during the experiment.

We performed two data acquisition experiments. Prior to data acquisition in each experiment, we decanted the gel into the container and started rotating the sample. The scanner was programmed such that the projections were acquired once the rotation stage reached the required rotational speed, which for the first experiment was 100 deg/s or 16.6 rpm. This experiment was performed such that 120 projections were collected over 360 degrees, for a total of 83 rotations, with exposure time 30 ms for each projection. This took a total of 5 min of acquisition time, during which the tablet moved due to saturation but did not completely dissolve. The spatial resolution was 95 μm, and the field of view was cropped to the boundaries of the sample holder (each projection image is of size 647 px × 768 px). The data are made available via [57]. Our experimental setup allowed the collection of open and closed shutter (flat- and dark-field) images before decanting the gel onto the tablet. No centrifugal force effect was observed on the bubbles travelling during the scan or in our test runs at the given rotational speed.

The collected data were streamed into Recast3D, where the three slices were visualised after the first 30 projections. We ran the Recast3D on a Linux machine with specs 2.1 GHz, 64-bit 32-core CPU (2x 16-core Intel Xeon Gold 6130) using a single GPU with 11 GB RAM (NVIDIA Geforce RTX 2080Ti). The delay from the moment data were collected to the update of the cross-sectional slices was minimal (under 0.002 s). The backprojection was performed by FDK [58] with Ramp filter on the slices in view. The cross-sections were refreshed every 30 projections (i.e., using the most recent 120 projections per backprojection), thus ensuring an update of sample dynamics every 0.9 s. This was done regardless of user interaction. Implementation details can be found in [8].

**Figure 14:**
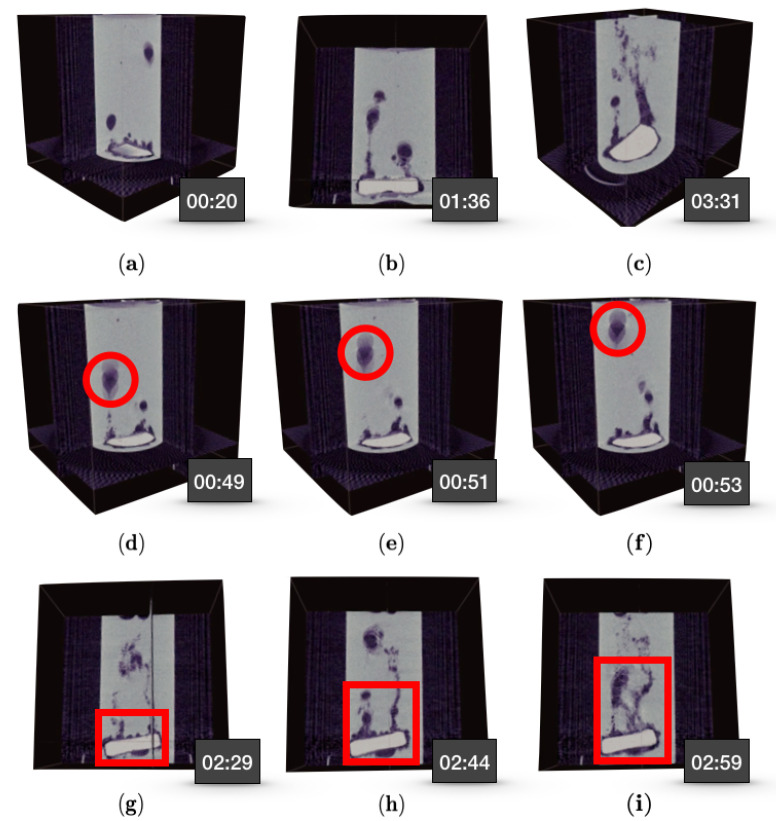
A collection of scenes from the live reconstruction: The top row shows the different interactions the user can perform during the live reconstruction (**a**–**c**); the middle row focuses on the dynamics of a single bubble travelling on a straight line (**d**–**f**), and the bottom row highlights the evolution of a trapped gas bubble underneath the tablet (**g**–**i**).

User interactions with Recast3D were recorded via a simple screen capture program. We also mounted an action camera on top of the source to record the full experiment. These items were brought together in a single video (aligned in time), which can be found in the Supplementary Materials. Figure 14 is a collections of scenes from the live reconstruction video, along with the acquisition timestamp. The top row (Figure 14a–c) indicates the interactions the user can perform during the live reconstruction: Figure 14a being the default view in Recast3D, Figure 14b the rotation about the centre of axes, and Figure 14c the tilting of the cross-sectional slices. For the middle row (Figure 14d–f), we adjust the view to allow tracking the dynamics of a single bubble, which is travelling on a straight line. We can deduce the speed at which this particular bubble is travelling from the time and displacement of the bubble. For the bottom row, we rotate the view to follow the evolution of a trapped gas bubble underneath the tablet, which first appears in Figure 14g, grows larger in Figure 14h, and finally is released in Figure 14i.

**Figure 15:**
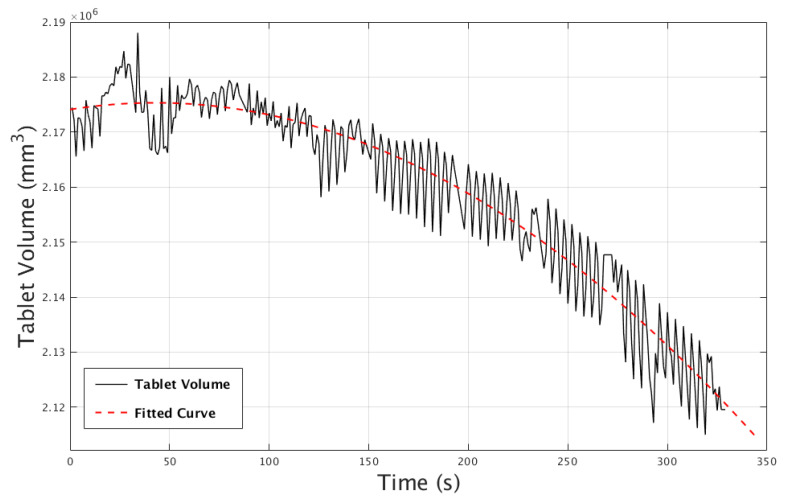
The decline in tablet volume over the 5-minute data acquisition period.

We further quantified the rate of dissolution of the tablet over the scan time (5 min) in the water-based gel. This was done by segmenting out the tablet at each point in time and calculating its volume. This is given in Figure 15, including a fitted curve to the data points. For the segmentation, we used a simple threshold to extract the tablet, and, for curve fitting, we used the tool provided as part of the Curve Fitting Tool [59] in MATLAB. The tablet volume data were fitted to a curve with the dips (due to severe artefacts in the segmentation) excluded. The graph initially shows no change in the tablet volume, and then a gradual decrease at a constant rate. We note that the oscillations in the experimental curve are due to tablet rocking up and down in the gel, and at times laying parallel with the rotational plane (the latter produces relatively stronger streak artefacts). These passed the threshold and were counted as part of the segmented volume at varying amounts, leading to the oscillations. These streak artefacts, along with the tablet moving during the scan, can be seen in the experiment video provided in the Supplementary Materials. According to the fitted model, the tablet would be completely dissolved in gel in approximately 27 min.

**Figure 16:**
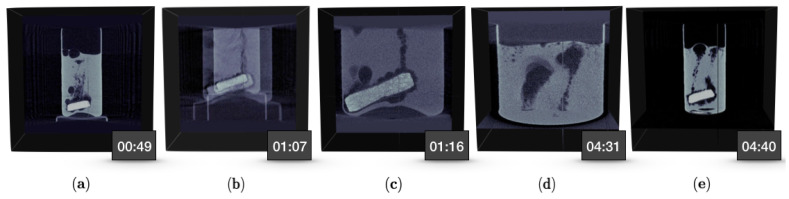
A series of frames from the live reconstructions, showing the initial magnification capturing the whole sample (**a**); the effect of zooming in during live reconstruction (**b**); reconstructions at higher magnification of the bottom part of the sample after the zoom (**c**); reconstructions at higher magnification of the top part of the sample after the shift in vertical axis (**d**); and zooming back out to the original magnification (**e**).

The experiment was repeated with the same setup except for a faster rotational speed and shorter exposure time, which were 200 deg/s (or 33.3 rpm) and 12 ms per projection, respectively. In this experiment, we also demonstrated a change in the geometry by zooming into the bottom of the sample at user’s command. We collected 150 projections per 360 degrees for a total of 166 rotations, which amounted to 5 min acquisition time (same duration as the initial experiment). Each projection was now binned but not cropped, i.e., each projection is of size 487 px × 385 px. The spatial resolution at the beginning of the experiment was 193 μm, which at an arbitrary point in the experiment changed to 76 μm. In this particular experiment, we initially zoomed in to the bottom of the sample to observe the tablet, and later moved the source and detector upwards to observe the surface of the sample. Since the sample holder was the same as the initial experiment, we had an idea of roughly where to zoom into and to what magnification. However, the up- or downward shifts in the vertical direction was decided on the spot, during the experiment. As in the initial experiment, we captured a video of the interior of the scanner via an action camera mounted on top of the source, and the user interactions with Recast3D via a screen capture program. Along with the collected radiographs, these videos were put together in multimedia files, available in the Supplementary Materials. Figure 16 shows a series of frames from the Recast3D video along with timestamps. The order of events was as follows: The data were collected as in the first experiment. At 01:06, we started to zoom in to magnification approximately 2.5× and reposition the source and detector to align with the bottom of the sample, i.e., the tablet. The initial zoom appeared in the visualisation until such a time the entire reconstruction was reinitialised (see Figure 16b). This is because, at user’s command, the motors first moved to the requested position and, when static again, a new geometry file was written to the data folder (indexed by the projection number, data are available in [60]). The detection of the new geometry file triggered a reinitialisation in the live view, and a new reconstruction was shown with a delay of 1.8 s (i.e., a full rotation). This was repeated for when the motors were moved upwards, albeit without the initialisation since no new geometry file was written. It took another 1.8 s for the reconstruction to fully stabilise, now showing the surface of the water-based gel (see Figure 16d). Before the end of 5 min duration, we zoomed out to the original view. When we zoomed into the tablet, we were able to observe the phenomenon mentioned in Figure 14g–i more clearly. In addition, when shifting the view to the surface of the gel, we could see the foaming of the effervescent bubbles on the top layer, which we did not observe in the initial experiment.

## 4. Discussions

Tomography is commonly used in many applications, and its use as a means of a research tool is rapidly growing. However, in many cases, the research question is not well defined prior to acquisition, and it is easy to overlook features or miss opportunities to investigate further without expert insight. This results in the under-utilisation of tomography as a tool in the traditional workflow. It is known that the quality of research depends heavily on the quality of acquired data. However, the sequential method of imaging offers no room for an immediate follow-up, leaving the expert to work around the data to fit the question, or to adapt the research to the data. This limits the potential of tomography as a tool and we argue it can be further utilised in many applications. The research question should not adapt to the data available. Instead, it should build up on them, learn from them and improve. In this paper, we offer an alternative that brings all aspects of tomographic imaging (from data acquisition to research analysis) into a single loop. This can be fine-tuned by the expert at any stage, and can be iterated until concrete results are achieved. We refer to this as explorative imaging.

We also introduce the FleX-ray laboratory, established in May 2017, located at CWI, Amsterdam, the Netherlands. This laboratory consists of a custom-built, highly flexible X-ray scanner, and the setup that aligns with the idea of explorative imaging. The framework outlined in Section 2 is used on a daily basis, with many external and internal users visiting the laboratory. The case studies in Section 3 were performed in-house with no additions or major changes made specifically for the experiments, and for each experiment the framework was followed naturally within our setup.

An overlook of the case studies highlights that the three components brought together in the FleX-ray Laboratory, namely the use of expert knowledge; the hardware flexibility; and the almost-instantaneous reconstructions (during and in-between scans), form the foundations of explorative imaging. A detailed look in all case studies reveals that the power of expert knowledge is evident, and often transforms the original research problem to a more clear question. This stems from the expert being able to consider any intermediate results to arrive at better posed questions, and therefore more accurate and detailed solutions. This is especially highlighted in the first case study in Section 3.1 with the further exploration with every loop in experimenting, and unexpected intermediate results allowing us to focus on regions that have more information than others. It was also due to the expert that we were able to pinpoint what was important or noteworthy in the intermediate reconstructions. This was also an important factor in Section 3.2 where the expertise helped us recognise the handwriting, which influenced the data collection and image processing, as well as filtering out the false positives otherwise difficult to detect.

The flexibility of the apparatus allowed us to focus on certain areas and to take radiographs we could later stitch, without having to physically or mechanically move or alter the object but instead command individual motors to go to a specified position in given direction. This is a big advantage in all study cases but especially beneficial in the second case study, as the object itself was very fragile and needed supports and testing for mounting alone. The flexibility of the scanner helped us bypass any possibilities of damaging the object or spending time remounting for each radiograph. This flexibility was useful in other cases for when we wanted to focus on certain regions such as the fingerprints in Section 3.1 (by tiling in detector plane) or in Section 3.3 to track bubbles (by computationally modifying perspective in 3D).

Perhaps the most unique aspect of performing the study in Section 3.3 at the FleX-ray Laboratory was the ability to see almost instantaneous slice reconstructions in three cross-sectional planes via Recast3D with the possibility of interaction and reaction during the scanning process by (in this case) zooming into regions of interest. This helped us monitor the effervescent bubbles in 3D as they travelled to the surface, or those that trapped escaped. We were able to quickly locate any events that were not expected or the locations unknown beforehand. In combination with the zooming, the ortho-views making up the quasi-volume, which can be moved or tilted, can be a powerful tool for dynamic experiments, in which a sample continuously undergoes structural changes, or in those that unexpected events may occur (object deformation or disintegration during acquisition).

### Our Vision for the Future

Explorative imaging paves the way to more benefits for research. With the setup introduced in this paper, the possibility of semi-autonomous X-ray imaging systems is the natural next step, where an operator and expert are assisted by algorithms (based on, e.g., heuristics, optimisation or even AI). We believe one can achieve such a system that can learn to make simple decisions based on the object size and desired spatial resolution. The system can adjust its motors to accommodate what is needed; decide whether there should be spatial tiling in detector or both detector and source planes; or adjust source settings automatically to return the optimal distribution of arriving photons. Further, the system can learn to highlight if something has changed in the object during the scan or readjust itself when requested to zoom in on a certain region.

In addition, the system can be improved to constantly output information about the acquisition or the object. This could be achieved via an on-the-spot segmentation or pre-defined physical quantification, which could be displayed during live reconstruction to best guide the expert when making decisions.

We believe that the proposed workflow in this paper, coupled with the learning to make simple decisions can further enhance the quality of data and therefore the research not only in CT but in other imaging modalities as well (for example, in EIT [61]).

## Data Availability

The data collected in the first experiment presented in Section 3.3 are made available via [57] and the second via [60]. The single and two-tile data acquired of “the Torso” (see Section 3.1) are available via [18].

## Supplementary Materials

The following are available online at https://zenodo.org/record/3738781#.Xoat3ju-mUl: We include videos of the action camera, radiographs and almost-real time reconstructions. Videos are put together as part of Section 3.3. In addition, we include various photographs of “the Torso” in Section 3.1 and of the manuscript in Section 3.2, as well as the uncorrected and unprocessed versions of the radiographs in Figure 12 and Figure 13.

## Software Information

Offline image reconstructions were done using MATLAB 2017b (Section 3.3, reconstructed to compile data for Figure 15) or TESCAN-XRE Recon (Section 3.1, reconstructions in Figure 4, Figure 5, Figure 6, Figure 7, Figure 8, Figure 9 and Figure 10, software provided as part of the FleX-ray Laboratory pipeline). Online image reconstruction in Section 3.3 was done via Recast3D. User interactions with Recast3D were recorded using SimpleScreenRecorder for Linux. The volume renderings in Section 3.1 were obtained using Drishti. Post-processing and image analysis were performed in MATLAB 2017b (Section 3.3) or ImageJ (Section 3.2). The image arrangement or annotation were done in Keynote. All schematics were prepared using Inkscape.
